# The Roles of Mitochondrial SIRT4 in Cellular Metabolism

**DOI:** 10.3389/fendo.2018.00783

**Published:** 2019-01-07

**Authors:** Zheying Min, Jiangman Gao, Yang Yu

**Affiliations:** ^1^Beijing Key Laboratory of Reproductive Endocrinology and Assisted Reproductive Technology and Key Laboratory of Assisted Reproduction, Ministry of Education, Department of Obstetrics and Gynecology, Center for Reproductive Medicine, Peking University Third Hospital, Beijing, China; ^2^Peking-Tsinghua Center for Life Sciences, Academy for Advanced Interdisciplinary Studies, Peking University, Beijing, China

**Keywords:** SIRT4, mitochondrial, enzymatic activities, insulin secretion, fatty acid metabolism, tumor suppressor

## Abstract

Sirtuins comprise a family of nicotinamide adenine dinucleotide (NAD^+^)-dependent lysine deacylases that regulate the life span, aging, and metabolism. Seven sirtuin family members (SIRT1-7) have been identified in mammals, including humans. Despite the indispensable role of mitochondrial sirtuin 4 (SIRT4) in metabolic regulation, the primary enzymatic activity of SIRT4 remains enigmatic. SIRT4 possesses ADP-ribosyltransferase, lipoamidase and deacylase activities. Interestingly, the enzymatic activities and substrates of SIRT4 vary in different tissues and cells. SIRT4 inhibits insulin secretion in pancreatic β cells and regulates insulin sensitivity as a deacylase in the pancreas. SIRT4 represses fatty acid oxidation (FAO) in muscle and liver cells differently. SIRT4 has also been identified as a mitochondrial-localized tumor suppressor. A comprehensive understanding of the enzymology of SIRT4 in metabolism is essential for developing novel therapeutic agents for human metabolic diseases. This review will update the roles of SIRT4 in cellular and organismal metabolic homeostasis.

## Introduction

**Figure 1:**
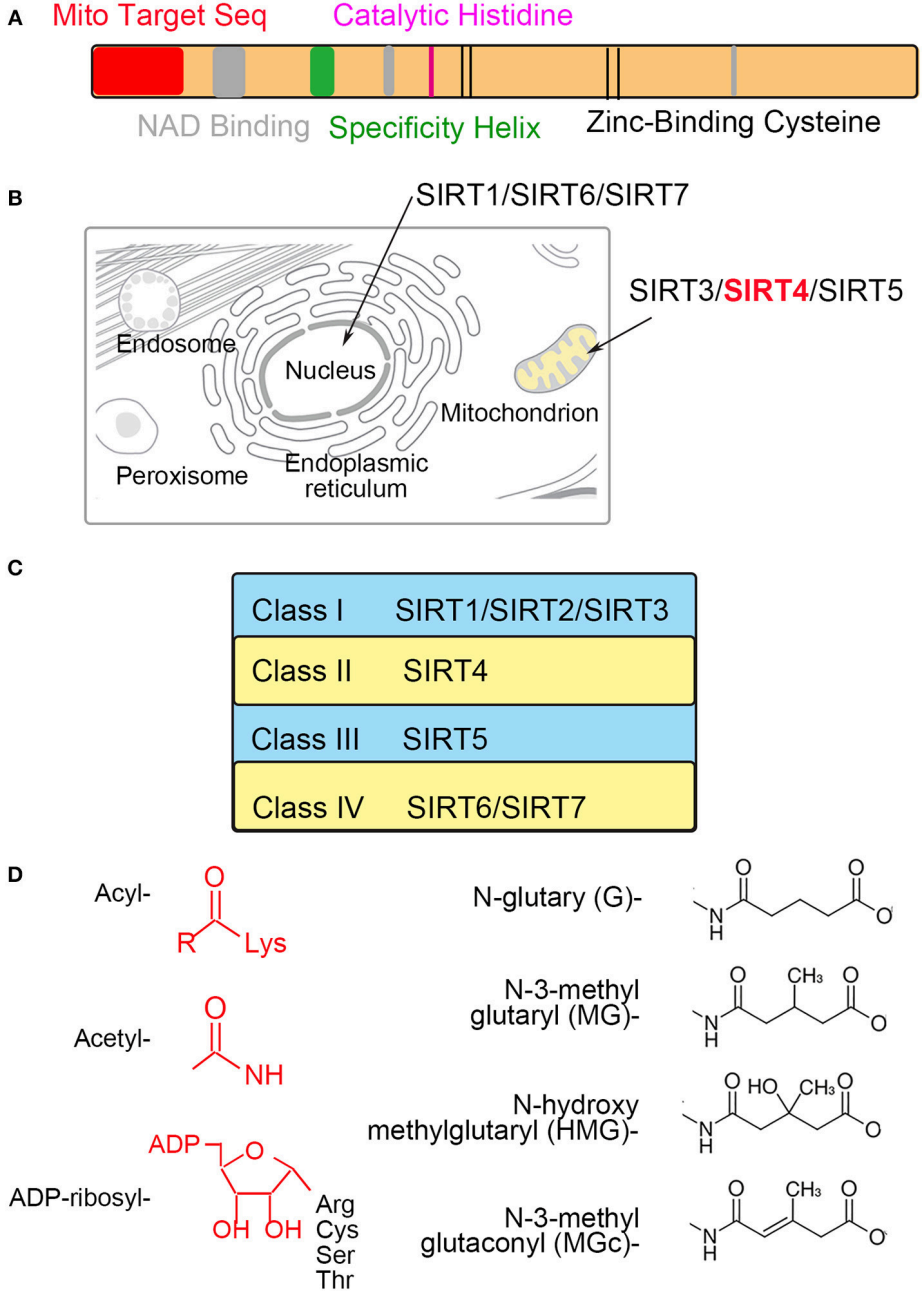
The characteristics of mitochondrial SIRT4 **(A)**. Sequence analyses show the amino acid sequence of the mitochondrial localization, NAD^+^-binding, substrate specificity, catalytic and Zn^2+^-binding domains **(B)**. SIRT4 is localized in the mitochondrion **(C)**. SIRT4 is belong to a class II human sirtuins **(D)**. Structure of groups catalyzed by SIRT4.

Sirtuins include β-NAD^+^-dependent deacylases and ADP-ribosyltransferases involved with metabolism and aging (1, 2). Sirtuins (SIRT1-7) can be divided into nuclear (SIRT1, SIRT6, and SIRT7), mitochondrial (SIRT3, SIRT4, and SIRT5) and cytosolic (SIRT2) forms, and some sirtuins are found in more than one compartment (Figure 1B). The mitochondrial sirtuins are involved in metabolic regulation and antioxidative defense. In contrast to other members of SIRT, the enzymatic activities of SIRT4 have remained unclear (3, 4). SIRT4 is critical for cellular metabolism and DNA damage responses in mitochondria. SIRT4 contains an N-terminal mitochondrial signal sequence that directs of its localization to mitochondria (4). SIRT4 has the requisite amino acids to participate in deacylase reactions (5), namely, a homologous sirtuin deacylase domain, a conserved catalytic histidine, and a Rossmann fold/NAD^+^-binding motif (aa 62–82, 143–146, 260–262, and 286–288) (Figure 1A). Previous genetic studies divide human sirtuins into four classes. SIRT4 belongs to the class II human sirtuins (Figure 1C). SIRT4 mRNA and protein are detected in human muscle, kidney, testis, and liver cells (6). This expression pattern is consistent with the functions of SIRT4. In this review, we summarize the roles of SIRT4 in cellular metabolic homeostasis, including the regulation of insulin secretion and fatty acid oxidation and the effect on tumor cells of different tissues (Table 1).

**Table 1:** Summary of roles of SIRT4 in celluar metabolism.

**Genotype**	**Phenotype**	**Process**	**Activities**	**References**
SIRT4 OE cells	Inhibition of insulin secretion in pancreas β cells	ADP-ribosylation and inhibition of GDH	ADP-ribosylation	(6–8)
SIRT4 KO cells	Activation of GDH and stimulation of insulin secretion in insulinoma cells	Activation of GDH	ADP-ribosylation	
SIRT4 KO mice	Increased insulin secretion,	Activation of GDH	ADP-ribosylation	
SIRT4 KO mice	Deregulated lecuine metabolism and aging induced IR	Dysregulated leucine oxidation	deacylation	(9–11)
SIRT4 OE cells	Increased lipogenesis and decreased FAO in adipocyte and myocyte cell	Deacetylation of MCD	deacetylation	(12–15)
SIRT4 KO cells	Increased FAO in muscle and WAT cells	Activation of MCD and decreased malony-CoA	Deacetylation	
SIRT4 KO mice	Elevated FAO and resistance to obesity and exercise	Activation of GDH	Deacetylation	
SIRT4 OE cells	Depressed FAO in liver cells	Repression of PPARα transcriptional activation by SIRT1	–	(12, 16–18)
SIRT4 KO cells	Increased FAO rate	Activation of PPARα	–	
SIRT4 KO mice	Increased FAO rate and PPARα pathway	Activation of FAO by PPARα and SIRT1	–	

OE, over expression; KO, knock out.

## SIRT4 Inhibits Insulin Secretion in Pancreatic β Cells

SIRT4 was initially found to reduce glutamate dehydrogenase (GDH) activity, thereby inhibiting insulin secretion in pancreatic β cells (7, 9). GDH is known to facilitate glutamine metabolism and ATP production, thus inducing insulin secretion (19, 20). GDH is ADP-ribosylated and inhibited by SIRT4, subsequently repressing leucine-mediated insulin secretion (7, 21). The depletion of SIRT4 in insulinoma cells could activate GDH, thus increasing amino acid-stimulated insulin secretion. Pancreatic β cells derived from SIRT4 knockout mice and those from mice on calorie restriction (CR) as a dietary regimen show a similar effect of insulin secretion (8, 22). Phosphodiesterase (PDE) can be used as a probe for ADP-ribosylation because it cleaves the ADP-ribose and can relieve inhibition. GDH from SIRT4-deficient mice is insensitive to (PDE), and incubation with PDE increases the GDH activity of wild-type lysates to the KO samples. It indicates the absence of the enzymatic cleavage of ADP-ribose would decrease ADP-ribosylation of GDH (6, 8).

**Figure 2:**
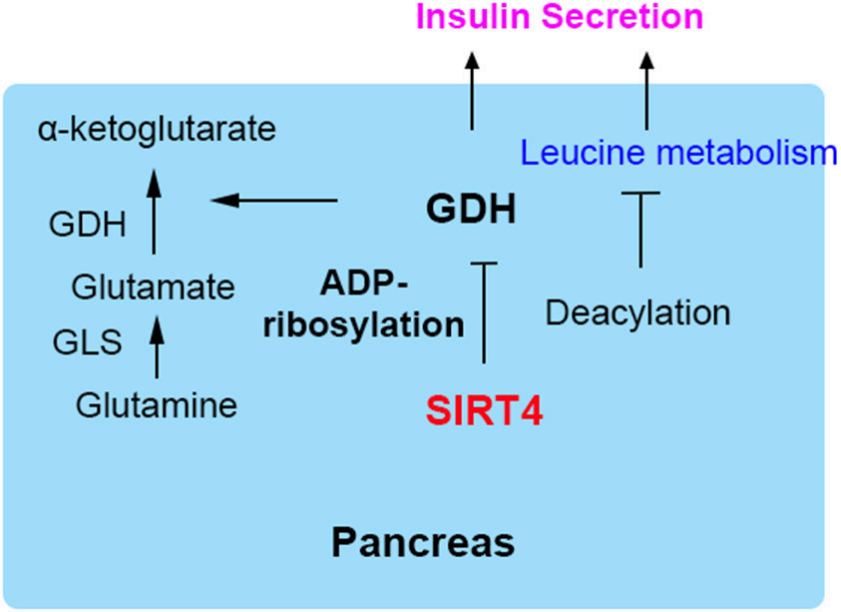
SIRT4 regulates insulin secretion in pancreatic β cells. GDH is ADP-ribosylated and inhibited by SIRT4, repressing insulin secretion.

A recent study demonstrated that SIRT4 catalyzed the removal of novel lysine modifications: methylglutaryl (MG)-lysine, hydroxymethylglutaryl (HMG)-lysine, and 3-methylglutaconyl (MGc)-lysine (10, 11). SIRT4 participates in leucine oxidation by removing these modifications sequentially. These modifications were discovered and characterized through phylogenetic and structural analysis (Figure 1D). The α-helical region containing the catalytic pocket of SIRT4 was associated with an interaction with negatively charged acyl- modifications (23, 24). In a complementary study, a recombinant SIRT4 protein could remove glutaryl-, MG-, HMG-, and MGc-lysine modifications. Further, SIRT4 overexpression in cells resulted in decreased glutaryl-, MG-, and HMG-lysine expression levels. The methylcrotonyl-CoA carboxylase complex (MCCC) associated with leucine catabolism and interacting with SIRT4 was marked with these new modifications *in vivo* (10). The absence of SIRT4 increased and destabilized MCCC acylation, leading to decreased leucine flux. The level of a key allosteric GDH activator, leucine, was changed due to the repression of GDH activity and glutamine metabolism by SIRT4 (25, 26). Knowledge about these new modifications provided new insights into SIRT4 function in further metabolism studies (Figure 2).

## SIRT4 Inhibits Fatty Acid Oxidation in Muscle and Fat Cells

**Figure 3:**
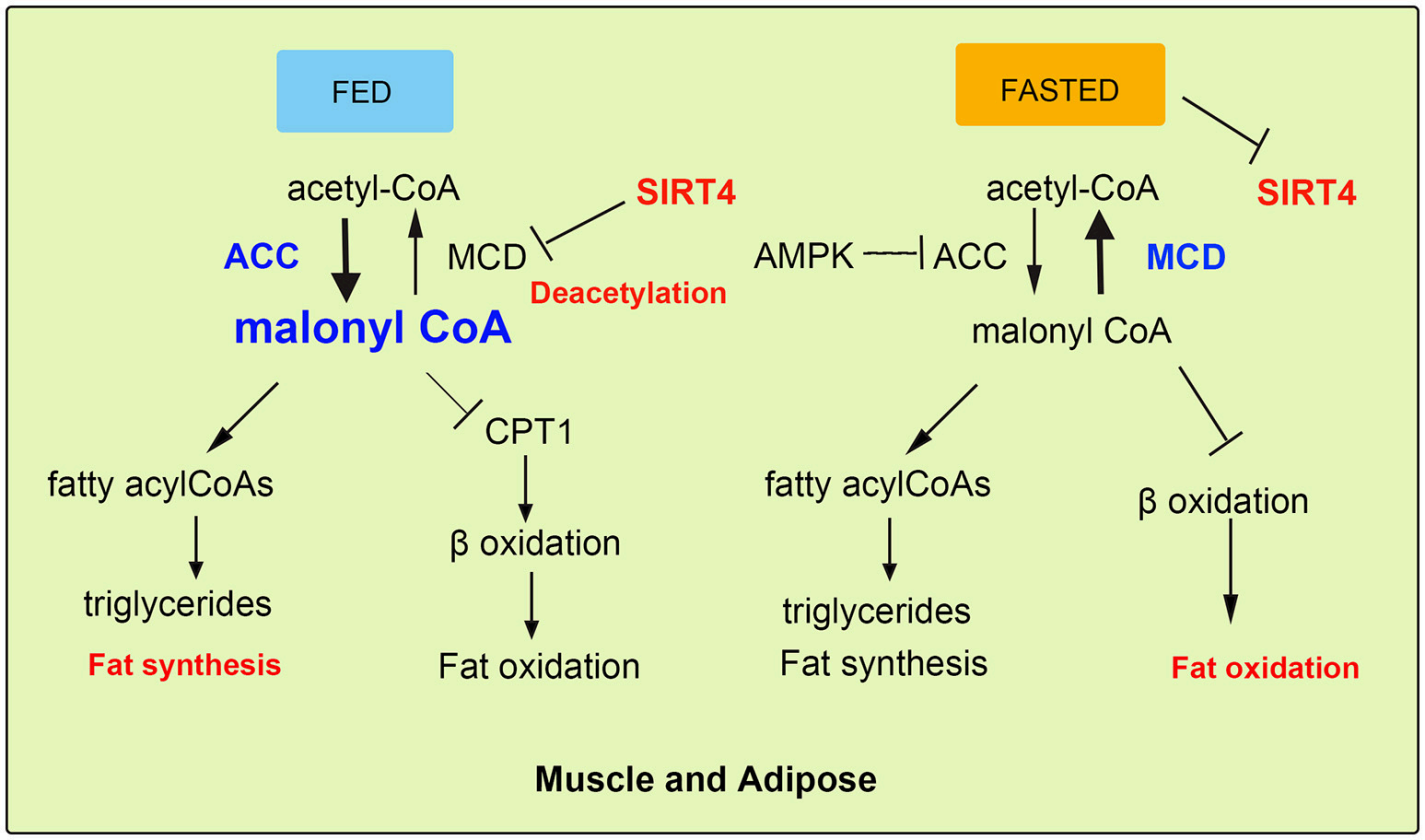
SIRT4 mediates fatty acid oxidation in muscle and adipose cells. In fed state, SIRT4 decreases the activity of the mitochondrial malonyl-CoA decarboxylase (MCD), which can increase malonyl-CoA levels, thus inhibiting FAO. In fasted state, the level malonyl-CoA of is decreased conversely.

The repressive effect of SIRT4 against FAO in muscle cells is regulated by the deacetylation and inhibition of the activity of mitochondrial malonyl-CoA decarboxylase (MCD), leading to an increase in malonyl-CoA (12, 13, 27). Malonyl CoA is a key metabolite that inhibits fat catabolism and promotes fat synthesis (28). There are two enzymes that regulate cellular malonyl-CoA levels. MCD catalyzes the conversion of malonyl-CoA to acetyl-CoA, while acetyl-CoA carboxylase (ACC) converts acetyl-CoA back to malonyl-CoA via a reaction regulated by the phosphorylation of AMPK (14, 29). Cytosolic fatty acids transported into the mitochondrial matrix cross the outer and inner mitochondrial membranes for β-oxidation, and this key step is catalyzed by carnitine palmitoyltransferase (CPT1), which is inhibited by accumulated malonyl-CoA (30, 31). During the nutritional rich state (Fed), when the metabolic intermediates funnel into fat synthesis and energy storage, the steady state is increased malonyl-CoA. This suppresses entry of fatty acids into mitochondria and weakens FAO sequentially (15). Conversely, in the fasted state, the malonyl-CoA is decreased and the FAO is evaluated for energy production (Figure 3). Therefore, SIRT4 regulates malonyl-CoA levels of muscle and WAT of mice in the fed and fasted state. In addition, as a unit of fatty acid synthesis, the accumulated malonyl-CoA promotes and provides a carbon skeleton for fatty acid biosynthesis in white adipose tissue (WAT) (32, 33). A previous study reported that SIRT4 overexpression increased lipogenesis and decreased fatty acid oxidation, while SIRT4 knockdown showed the opposite effect on lipid synthesis and catabolism in mouse adipocyte and myocyte cell lines (3, 34). Furthermore, the regulation pattern was confirmed in SIRT4 knockout mice, which showed elevated FAO associated with a resistance to diet-induced obesity and an increased exercise tolerance (24, 35, 36). Additionally, the interaction of MCD and SIRT4 in the mitochondrial matrix was demonstrated by coimmunoprecipitation. SIRT4 lacked detectable histone deacetylase activity, but it deacetylated MCD in a substrate-specific manner (37, 38). Further, mitochondrial located SIRT3 was confirmed and excluded the possibility of deacetylation activity, because interaction between SIRT3 and SIRT4. SIRT3 showed no detectable interaction with MCD indicating the specific function of SIRT4. SIRT4 coordinated lipid homeostasis by promoting lipid anabolism and repressing lipid catabolism (39, 40).

## SIRT4 Represses Hepatic FAO in Liver Cells

Liver plays a role in energy homeostasis by balancing lipid metabolism and energetic demands in organisms (12, 16). Under nutrient-rich conditions, hepatic lipogenesis, and lipoprotein secretion are activated. In contrast, during fasting, hepatic FAO is stimulated to provide the organism with ketone bodies as cellular energy fuel for the brain. One of the key mediators of the hepatic response to fasting is nuclear receptor peroxisome proliferation-activated receptor α (PPARα). SIRT4 negatively mediated fatty acid oxidation in liver cells by suppressing the transcriptional activity of PPARα (17, 41). More specifically, the interaction of SIRT1 and PPARα was disrupted by SIRT4, thus attenuating the activation of PPARα transcriptional activity via SIRT1 to inhibit FAO (18). In normal liver cells, by interacting with PPARα, SIRT1 is recruited to the PPARα response element (PPRE), which catalyzes the N^ε^-acetyl-lysine deacetylation of the transcriptional coactivator peroxisome proliferator-activated receptor gamma coactivator 1-alpha (PGC-1α) to promote FAO (42). Additionally, SIRT4 competes with other sirtuins including SIRT1 for β-NAD^+^, leading to decreased SIRT1 activity and a reduced effect of SIRT1 on both the transcriptional activity of PPARα and FAO (43, 44). During fasting, FAO is promoted as a source of cellular energy. The decreased SIRT4 levels in liver cells demonstrate the inhibitory effect of SIRT4 on liver FAO (45).

**Figure 4:**
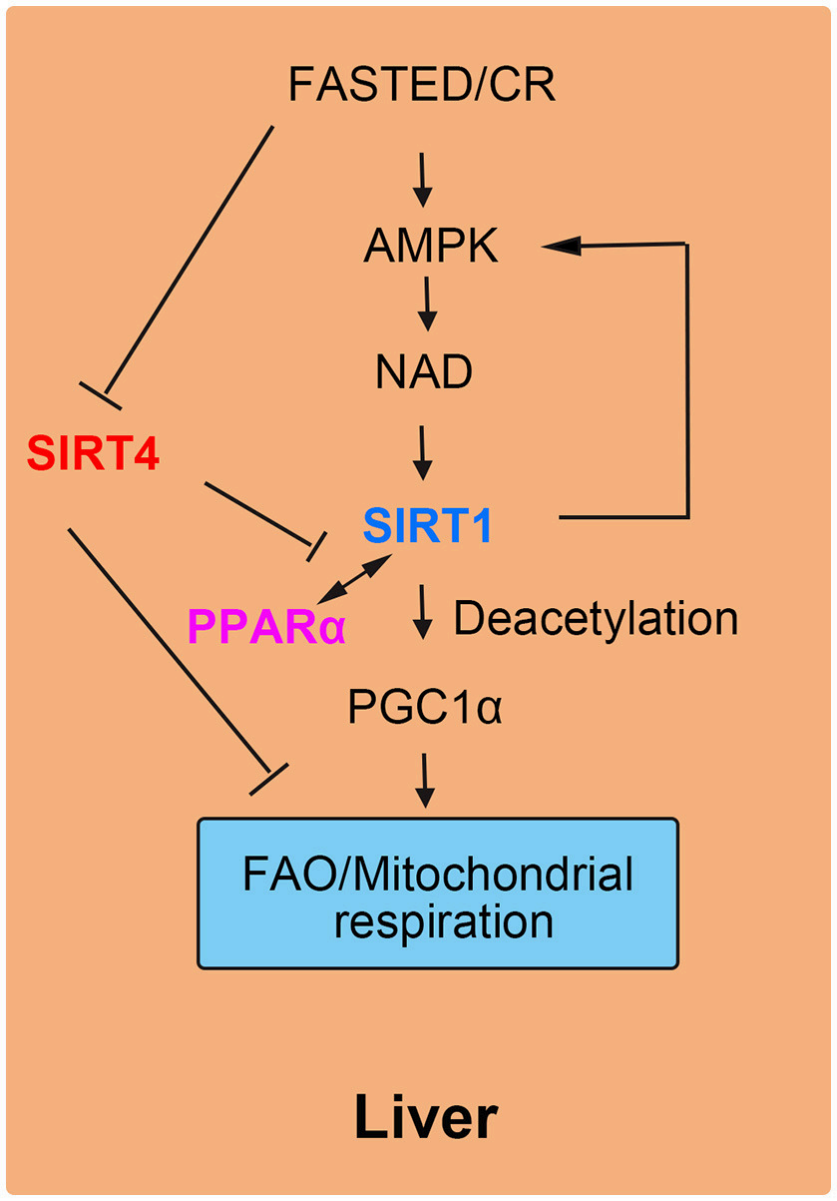
SIRT4 mediates fatty acid oxidation in liver cells. The interaction of SIRT1 and PPARα is blocked by SIRT4, leading to a decrease in FAO.

The roles of SIRT4 in liver cells were supported by knockout and overexpression experiments in cellular and animal models. In SIRT4 knockdown and knockout primary hepatocytes, the expression of mitochondrial and fatty acid metabolism enzyme genes was increased significantly (17). Consistent with increased FAO gene expression, SIRT4 knockdown hepatocytes exhibited higher rates of FAO than wild-type cells. The increase in FAO gene expression in SIRT4 knockout mice was consistent with the results in primary hepatocytes (12). Additionally, SIRT1 mRNA and protein levels were also evaluated both *in vitro* and *in vivo* by the knockdown of SIRT4. In SIRT4/SIRT1 double-knockdown hepatocytes, there was not an increase in FAO compared with that induced by SIRT4 knockdown only (17). These results suggested that SIRT4-modulated FAO was dependent on SIRT1 in primary hepatocytes (22, 46). Moreover, SIRT4 overexpression repressed SIRT1 activation of PPARα and consequently inhibited hepatic FAO (Figure 4).

## SIRT4 Participates in Cellular ATP Homeostasis

In addition to regulating FAO, SIRT4 was shown to contribute to cellular ATP homeostasis and mitochondrial biogenesis. Deletion of SIRT4 decreased ATP levels, and overexpression of SIRT4 increased ATP levels (3, 47). ATP/ADP translocase 2 (ANT2a, transmembrane protein spanning the inner mitochondrial membrane) helped SIRT4 mediate cellular ATP homeostasis. Mechanistically, ANT N^ε^-acylation facilitated mitochondrial respiration by enhancing mitochondrial uncoupling and decreasing ATP production (48). Therefore, SIRT4 catalyzed the N^ε^-acyl-lysine deacylation of ANT2 to reduce mitochondrial uncoupling, leading to enhanced ATP production. In the mitochondrial signaling pathway, the depletion of SIRT4 activated AMPK to decrease ATP levels. Activated AMPK phosphorylates and inhibits cytosolic ACC, leading to a reduction in MCA and the promotion of FAO as mentioned above.

## SIRT4 Acts as a TUMOR Suppressor

SIRT4 is necessary for the regulation of FAO in normal cells but is also identified as a mitochondrial-localized tumor suppressor (49) (Table 2). SIRT4 has been recognized to possess tumor-suppressive effects due to the crucial regulatory role of mitochondrial metabolism in tumourigenesis (34, 50).

**Table 2:** Summary tumor repressor roles of SIRT4.

**Phenotype**	**Type of tumor**	**Roles**	**References**
SIRT4 is decreased	Acute myeloid leukemia	Inhibiting proliferation of tumor	(50, 51)
SIRT4 is decreased	Burkitt lymphoma	Inhibiting proliferation of tumor	(52, 53)
SIRT4 is decreased	Colorectal cancer	Inhibiting proliferation and migration of tumor	(54, 55)
SIRT4 is decreased	Gastric cancer	Inhibiting proliferation and migration of tumor	(56)
SIRT4 is decreased	Lung tumor	Inhibiting proliferation and migration of tumor	(57, 58)

SIRT4 plays an important role in the response to DNA damage by regulating the DNA damage-induced inhibition of glutamine catabolism (4, 57, 59). DNA damage increases the flux through the pentose phosphate pathway and decreases glutamine uptake and the levels of TCA cycle intermediates. Mitochondrial glutaminase can catabolize glutamine to form glutamate via mitochondrial GDH and aspartate aminotransferase activity (60). Previous research showed that SIRT4 ADP-ribosylated and inhibited GDH and was thus involved in DNA damage-induced inhibition of glutamine metabolism and anaplerosis (33, 39).

SIRT4 possesses a tumor suppressive effect because of its inhibitory effect on glutamine catabolism and its antiproliferative effect on cells with DNA damage (61, 62). Indeed, SIRT4 expression is upregulated by DNA damage and is downregulated in many types of human tumor tissues and cells (44). SIRT4 depletion leads to both elevated glutamine-dependent proliferation and stress-induced genomic instability, resulting in tumourigenic phenotypes. SIRT4 inhibits glutamine catabolism, especially replenishment into the citric acid cycle, creating a cellular state promoting DNA damage repair (63). In the absence of SIRT4, DNA damage results in delayed DNA repair and increased chromosomal aneuploidies, suggesting that SIRT4 could protect cells from spontaneous damage. Furthermore, SIRT4 null mice spontaneously develop lung tumors (19, 64).

**Figure 5:**
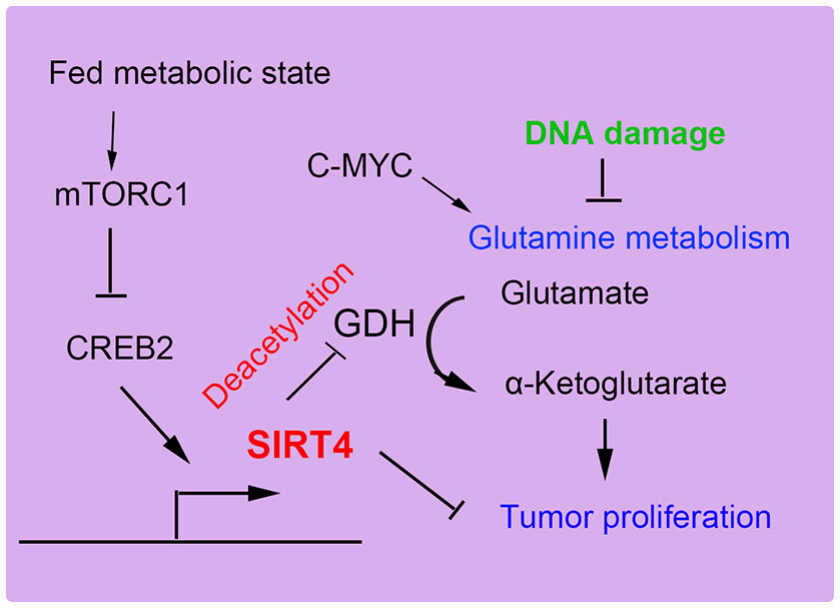
SIRT4 plays a role in tumor suppression. SIRT4 suppresses tumor proliferation by inhibiting glutamine metabolism and DNA damage repair.

SIRT4 can specifically repress the growth of B cells induced by the transcriptional factor c-Myc through inhibiting glutamine metabolism induced by the abnormal activation of c-Myc in c-Myc-dependent cancers (52, 65). In human Burkitt lymphoma cells, the overexpression of SIRT4 repressed glutamine metabolism and glutamine-dependent cell proliferation, as observed in cells treated with glycolysis inhibitors, promoting cell death (66). Consistent with these findings, the depletion of SIRT4 resulted in increased glutamine-absorbing and GDH enzymic activity in a mouse model of Burkitt lymphoma induced by c-Myc dysregulation. Additionally, SIRT4 suppressed Burkitt lymphoma driven by c-Myc, independent of c-Myc activity. Furthermore, SIRT4 was found to be overexpressed in colorectal cancer cell lines and to increase E-cadherin expression, which resulted in the suppression of cell proliferation and invasion (59). During the development of colorectal cancer invasion, SIRT4 expression was decreased. The suppressive role of SIRT4 is also mediated by inhibiting glutamine metabolism in colorectal cancer (54). Moreover, SIRT4 could enhance the sensitivity of colorectal cancer cells to the chemical drug 5-fluorouracil by inhibiting the cell cycle, thus showing the antiproliferative effect of SIRT4 overexpression (55). Recent reports have demonstrated that mammalian target of rapamycin complex1 (mTORC1) is correlated with increased nutrient uptake and metabolism. mTORC1 promotes glutamine supplement by activating GDH required transcriptional repression of SIRT4. Specifically mTORC1 suppresses SIRT4 by destabilizing of cAMP-responsive element binding 2 (CREB2) (67). Overexpression of SIRT4 decreases cell growth, transformation and development of tumor. Furthermore, leucine is an important positive regulator of mTORC1, which activity may be regulated by SIRT4 by reducing intracellular leucine levels (Figure 5). Therefore, as a glutamine steward, SIRT4 acts as a necessary component of the DNA damage response pathway that manages the metabolic blockade of glutamine metabolism, the cell cycle, and tumor suppression.

## Conclusion

**Figure 6:**
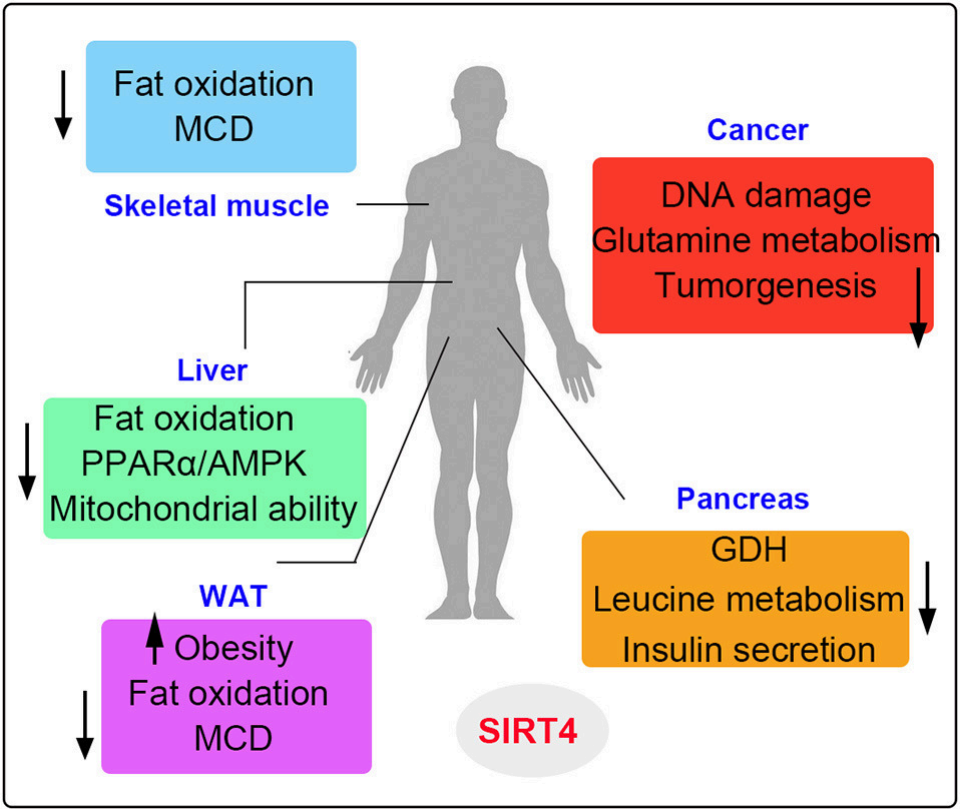
The metabolic roles of SIRT4 in various organs. SIRT4 is mainly involved in the metabolism of muscle, liver, fat, pancreatic, and cancer cells. The up arrows indicate the increased regulation pathways and the down arrows indicate the decreased regulation pathways induced by SIRT4.

In this review, we summarize different roles of SIRT4 in terms of enzymatic activities and functions in specific tissues and cells. SIRT4 possesses ADP-ribosyltransferase, lipoamidase and deacylase activities (46, 68, 69). Importantly, the enzymatic activities, and substrates of SIRT4 vary in different tissues and cells (Figure 6). SIRT4 inhibits insulin secretion as an ADP-ribosyltransferase and regulates insulin sensitivity as a deacylase in the pancreas. SIRT4 represses fatty acid oxidation (FAO) in the muscles and livers differently. SIRT4 has also been identified as a mitochondrial-localized tumor suppressor. SIRT4 is a less well-understood mammalian sirtuin, its cellular functions require further discovery. Knowledge of roles SIRT4 in cellular metabolism is helpful to provide new insights in the potential development of therapeutic agents for human diseases by targeting enzymatic activities (36).

## Author Contributions

ZM and JG draft the original manuscript. YY proofed and edited the finalized manuscript.

### Conflict of Interest Statement

The authors declare that the research was conducted in the absence of any commercial or financial relationships that could be construed as a potential conflict of interest.
